# Comparative Evaluation of Smartphone‐Based Fundus Imaging Systems in Dogs and Cats

**DOI:** 10.1111/vop.70210

**Published:** 2026-06-11

**Authors:** Bertrand Michaud

**Affiliations:** ^1^ Clinique Vétérinaire Anima‐Vet Saint‐Genis‐Pouilly France

**Keywords:** android, Aurora, cats, clearview, dogs, nun, Panoptic iExaminer, smartphone in ophthalmology iPhone, smartphone‐based fundus imaging, VistaView

## Abstract

**Objective:**

To compare the feasibility, image quality, acquisition time, and evaluator preference of four smartphone‐based fundus imaging (SBFI) systems in dogs and cats using a handheld fundus camera as a reference device.

**Animals Studied:**

Twenty client‐owned animals, including 10 dogs and 10 cats.

**Procedures:**

Fundus images were obtained from 40 eyes (20 mydriatic and 20 non‐mydriatic) using four SBFI systems (iExaminer Panoptic, NUN WFE‐02S, OQVet, and VistaView) and a handheld infrared fundus camera (Aurora). Image acquisition success and acquisition time were recorded for each device. Image quality was independently graded using a 10‐point Likert scale by a masked panel of 32 veterinary ophthalmologists. Evaluators also selected their preferred image among the four SBFI systems.

**Results:**

Image acquisition was successful in all animals for all devices under both mydriatic and non‐mydriatic conditions (100%). Image quality differed significantly between devices. Among SBFI systems, OQVet achieved the highest median score (7; interquartile range [IQR] 6–8), followed by VistaView (6; IQR 5–8), NUN (5; IQR 4–7), and iExaminer (4; IQR 3–5). OQVet received approximately 50% of evaluator preference votes. The reference camera achieved the highest image quality in 77.5% of imaging sets but was outperformed by OQVet (15%) and VistaView (7.5%) in some examinations. Pupil dilation did not significantly influence image quality or acquisition time.

**Conclusions:**

Smartphone‐based fundus imaging enables reliable retinal imaging in dogs and cats and represents an accessible option for retinal documentation and screening in veterinary practice.

## 1 Introduction

Fundus photography is one of the widely used imaging modalities used in ophthalmology for the screening, diagnosis, documentation, teaching, and longitudinal monitoring of retinal and optic nerve disorders [1, 2].

In human medicine, fundus photography has become an essential component of routine eye care, supported by the widespread availability of high‐quality tabletop and handheld retinal cameras. In contrast, veterinary ophthalmology faces specific constraints: human fundus cameras are not adapted to animal patients in terms of ergonomics and positioning, and their cost often precludes routine acquisition by veterinary clinics [3]. As a result, there is a growing demand for portable, easy‐to‐use, and inexpensive retinal imaging systems suitable for companion animals.

With the ubiquity of smartphones in daily life, their integration into healthcare applications has expanded rapidly. Among the various medical uses of smartphones—including patient education, data collection, and teleconsultation—smartphone‐based fundus imaging (SBFI) represents one of the most technically advanced and clinically promising applications [4, 5].

Early attempts at smartphone fundoscopy relied on indirect ophthalmoscopy using a handheld condensing lens aligned with the smartphone camera and light source, although direct approaches without a condensing lens have also been described. While these techniques demonstrated feasibility, they require precise alignment, significant operator skill, and may be limited by pupil size, often necessitating pharmacological mydriasis. In addition, images are typically inverted, similar to those obtained with indirect ophthalmoscopy using universal three‐dimensional (3D)–printed lens adapters [6, 7].

More recently, some fundus cameras attached to smartphones were developed and used similarly to a direct ophtalmoscope (D‐EYE digital ophtalmoscope, D‐EYE Srl, Padova, Italy). Although these devices improved ease of use, they were limited by a restricted field of view and compatibility with a narrow range of smartphone models, resulting in variable and often suboptimal image quality [8, 9].

Recent technological advances have enabled the development of compact, low‐power, and relatively inexpensive smartphone‐based retinal imaging systems. These newer Smartphone‐Based Fundus Imaging (SBFI) devices appear to offer a good alternative to conventional digital fundus photography [2, 10].

Newer SBFI utilize a small form factor, allowing for greater portability and ease of use. SBFI allows for a mobile fundus examination, is applicable both with and without pupil dilation, comes with built‐in connectivity and post‐processing capabilities, and is relatively easy to master. These handheld cameras are available at significantly lower service costs and range from $ 700–3000 [10, 11].

Importantly, SBFI systems can be delegated to trained technicians or paramedical staff, making them well suited for telemedicine applications and educational use, and potentially serving as surrogates for direct ophthalmoscopy in both human and veterinary settings [5, 12, 13]. However, multiple studies in human ophthalmology have demonstrated substantial variability in image quality between different smartphone‐based systems, underscoring the need for comparative evaluations and reference standards [10, 14, 15].

In veterinary medicine, the literature on SBFI remains limited. Several feasibility studies have demonstrated that fundus photography and videography can be performed in dogs and cats using smartphone‐based systems, including custom or non‐patented adapters [7, 16]. More recently, dedicated smartphone‐based digital fundus cameras have been evaluated for screening of retinal and optic nerve disorders in companion animals, with encouraging results regarding feasibility and clinical utility [17].

Nevertheless, to date, no comparative study has systematically evaluated multiple commercially available smartphone‐based retinal imaging systems under standardized clinical conditions in veterinary ophthalmology.

In our study, we compared four smartphone‐based portable retinal imaging systems currently available on the market. We deliberately focused on devices that rely on the smartphone's built‐in camera rather than systems using a dedicated retinal camera displayed on a smartphone screen (like Remidio FOP or Wikioptics NUN+). The devices were assessed for feasibility of image acquisition, image quality, acquisition time, and evaluator preference, using a handheld fundus camera (Optomed Aurora) as a reference standard.

## 2 Material and Method

### 2.1 Study Population

Recruitment and data collection were carried out at Clinique Vétérinaire AnimaVet (Saint‐Genis‐Pouilly, France) between November and December 2025. A total of 20 client‐owned animals, including 10 dogs and 10 cats, were prospectively enrolled in the study. All fundus image acquisitions were performed by a single examiner to ensure consistency across all tested devices and imaging conditions.

Only animals with transparent ocular media were included. Media clarity was confirmed by a complete ophthalmological examination performed using slit‐lamp biomicroscopy (SL‐17, Kowa, Japan). Animals were excluded if corneal, lenticular, or vitreous opacities interfered with adequate fundus visualization.

The study population included animals with both normal and pathological retinas. Overall, 40 eyes were examined (one mydriatic eye and one non‐mydriatic eye per animal), of which 26 retinas were classified as clinically normal and 14 as pathological based on funduscopic findings. Retinal abnormalities included lesions consistent with hypertensive retinopathy, inflammatory and other non‐inflammatory retinal disorders. Demographic characteristics, including age, sex, neuter status, and retinal status by species, are detailed in Table 1.

**TABLE 1:** Study population by species.

Variable	Dogs	Cats
Animals (*n*)	10	10
Age (years, mean ± SD)	5.4 ± 7.2	8.2 ± 7.6
Sex (Male/Female)	4/6	5/5
Neutered (Yes/No)	8/2	9/1
Retina (healthy/pathological)	14/6	12/8

*Note:* Demographic characteristics of the canine and feline populations included in the study. Age is expressed in years (mean ± SD). Retinal pathology is reported per eye; an eye was considered pathological if classified as such in any imaging condition.

Informed consent was obtained from all animal owners prior to inclusion. The study protocol was approved by the local research and ethics committee (Comité d'éthique de l'École nationale vétérinaire de Lyon). All procedures complied with Directive 2010/63/EU and adhered to the ARVO Statement for the Use of Animals in Ophthalmic and Vision Research.

### 2.2 Imaging Modalities

**FIGURE 1:**
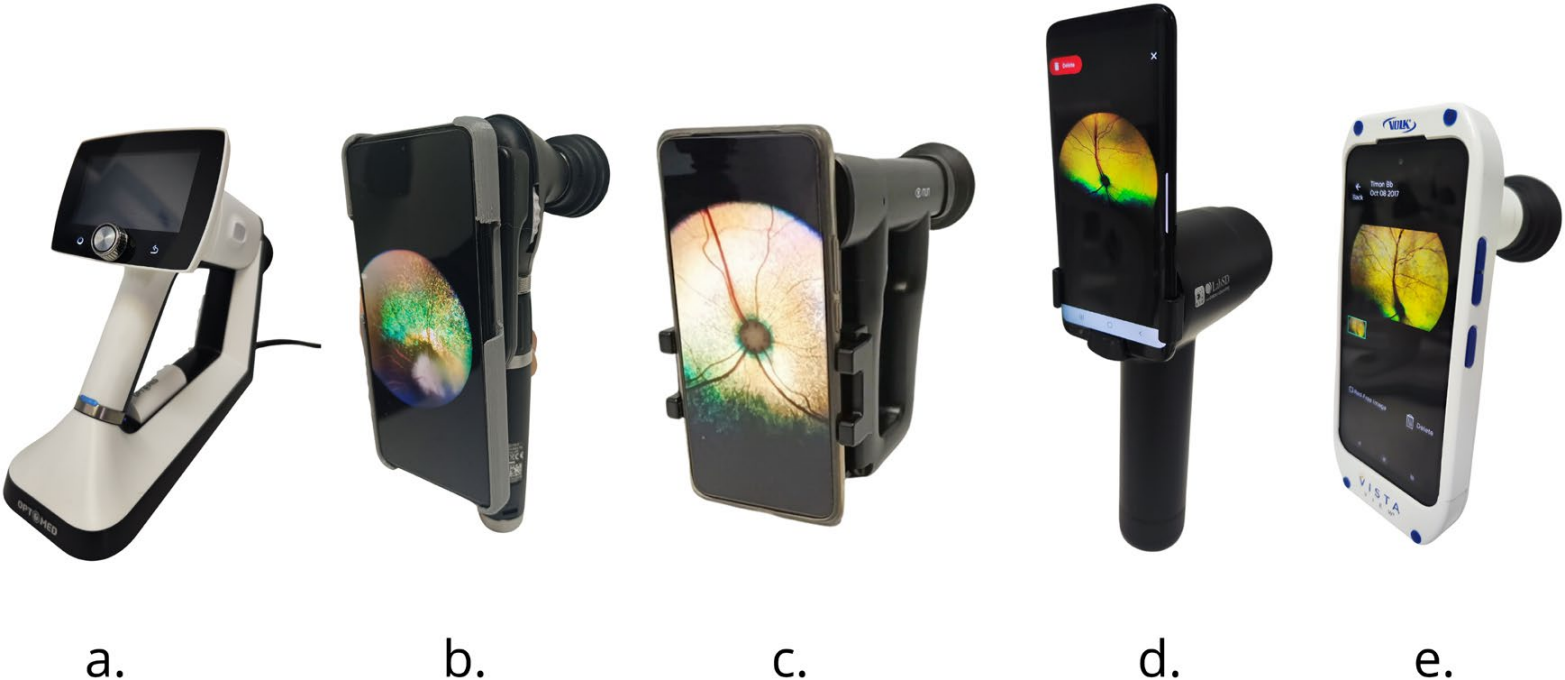
Fundus imaging devices evaluated in this study. Photographs of the reference handheld fundus camera (a) Optomed Aurora and the four smartphone‐based fundus imaging (SBFI) systems used in this study (b) iExaminer for Panoptic, (c) NUN WFE‐02S, (d) OQVet and (e) VistaView.

Four handheld smartphone‐enabled (Panoptic Iexaminer, Wikiopics NUN WFE‐02S, LabSD OQVet & Volk VistaView) retinal imaging device were compared with a non‐mydriatical Infra‐red fundus camera (Optomed Aurora) (Figure 1).

In the following subsections, we present the details about the publicly available smartphone‐based portable retinal imaging systems, the Aurora reference device and their features. Table 2 summarizes and compares the important features of Aurora and smartphone‐based retinal imaging systems publicly available in the market including their price, size, weight, compatible smartphones, illumination source, pupil dilation dependency, and degree of retinal view.

**TABLE 2:** Technical specifications and key characteristics of the smartphone‐based fundus imaging systems evaluated in this study.

	iExaminer	NUN	OQVet	Vistaview	Aurora
Compatible Smartphones	Universal	Universal	Galaxy S20	Nokia XR20, built in	N/A
Illumination Source/Type	Inside/Point	Inside/Ring	Inside/Flood	Inside/Flood	IR aiming/Flood
Dilation dependency	Not Required	Not Required	Not Required	Not Required > 4mm	Not Required
Degree of Retinal View (°)	25	30	60	55	50
Working distance (mm)	22	15	20	15	20
Size (mm)	70/220/162	125/42/155	81 × 160 × 198	88/202/103	122/202/258
Weight (g) with smartphone	390	365	390	310 (560) () with smartphone	853
Price ($)	1200	700	3600	1815	8000

The Optomed Aurora (Oulu, Finland) is a non‐mydriatic fundus camera with posterior (retinal) and anterior imaging modules. For the purpose of this study, only the retinal imaging module was utilized to visualize the fundus. The device utilizes a 50° FOV. We also utilized the rubber cup that comes with the device to reduce the impact of extraneous light during image acquisition. At the time of this study, the cost of the device was approximately $ 8000. In this study we will refer to this imaging modality as the Aurora (Figure 1a).

iExaminer is developed by attaching a smartphone to a Welch Allyn PanOptic Plus ophthalmoscope (Auburn, New‐York, USA). In the iExaminer system, the eyecup of the ophthalmoscope is removed and the smartphone is attached using the universal iExaminer SmartBracket to capture the retina images using the smartphone's camera. The iExaminer system involves three main pieces, including a smartphone application, iExaminer adapter, and PanOptic Ophthalmoscope. iExaminer provides up to 25° FOV for dilated eyes and allows adjusting focus ranging from −20 to +20 diopter. Its optic design generates its own light, converges it to a point at the cornea, and diverges around the retina. This allows easy entry into small pupils and wide area illumination of the fundus. Therefore, it may not require pupil dilation for retinal imaging. The operator can also control the amount of illumination manually. At the time of this study, the cost of the device was approximately $ 1200. In this study we will refer to this imaging modality as the iExaminer (Figure 1b).

WikiOptics Nun Ophtalmoscope WFE‐02S (Yongin, Gyeonggi, South Korea) is a brand new ophtalmoscope allowing a 30° FOV when associated with a universal smartphone adapter and the Nun application. It consists of a lens, the relay lens, and an ocular. It has a built‐in ring illumination system with a dimmer, two color filters (cyan and blue) as well as manual focus adjustment from −20D to +20D. A rubber cup comes with the device to reduce the impact of extraneous light during image acquisition. At the time of this study, the cost of the device was approximately $ 700. We'll refer to this device as the Nun (Figure 1c).

The OQVet (Lab SD, South Korea) is a smartphone‐based digital ophthalmoscope developed for veterinary retinal imaging (Figure 1d). The device provides a 60° field of view and is primarily designed for use under mydriatic conditions. It relies on a Galaxy S20 smartphone, supplied as part of the system, configured with optimized imaging settings for fundus acquisition.

The optical design integrates an internal illumination system and a dedicated imaging pathway aligned with the smartphone camera sensor. The device is powered by four AA batteries and includes a Bluetooth‐connected remote shutter control to facilitate image capture.

Images can be stored locally on the smartphone in stand‐alone mode or uploaded to a cloud‐based platform, depending on user preference. The system can function independently as a portable retinal imaging device or be integrated into a broader tele‐ophthalmology framework, enabling remote image review and consultation.

The relatively wide field of view compared with other smartphone‐based systems evaluated in this study represents one of its distinguishing technical characteristics.

Volk VistaView (Mentor, Ohio, USA) is a smartphone‐based fundus imaging device that has a veterinary mode to allow for imaging of the tapetum lucidum in animal eyes. The smartphone is a Nokia XR20 built in the device (and cannot be removed) and its camera takes the pictures but light is originating from an illumination board Bluetooth connected to the smartphone. A rubber cup is integrated with the device. The VistaView can image pupils as small as 4 mm and provides a 55° of retinal visualization. The user can control the brightness level of the device, change the focus and even voice activation picture capture through the VistaView App. the cost of the device was $1815 (including the smartphone). In this study, we will refer to this imaging modality as VistaView (Figure 1e).

### 2.3 Participant Imaging

Ten cats and 10 dogs were prospectively recruited to evaluate the four SBFI devices for their success/failure rates of image acquisition in non‐mydriatic and mydriatic settings.

Pharmacological mydriasis was induced in the right eye only of each participant by instilling one drop of tropicamide 0.5% (Mydriaticum, Laboratoires Théa, Clermont‐Ferrand, France) 20 min prior to imaging, in accordance with the manufacturer's recommendations. The contralateral eye remained non‐dilated, allowing intra‐individual comparison between non‐mydriatic and mydriatic settings.

Fundus photography was performed in a darkened examination room to minimize extraneous light, reduce lens reflections, and enhance physiological pupil dilation. All devices were directed toward the posterior pole to include the optic nerve head and surrounding retinal structures. Exposure parameters were adjusted depending on whether imaging targeted the pigmented non‐tapetal region or the reflective tapetum lucidum. Illumination intensity was systematically reduced when photographing the tapetal area to prevent overexposure.

For all smartphone‐based systems, image acquisition was performed using video mode. Videos were recorded at 4 K resolution and 60 frames per second. The best‐quality still frames were subsequently extracted after review. No software‐based image enhancement was applied other than standardized cropping.

A Xiaomi 12 Pro smartphone (112‐megapixel camera) was used with the universal SBFI adapters (iExaminer and NUN). VistaView incorporated a built‐in Nokia XR20 smartphone. The OQVet system was used in combination with a Samsung Galaxy S20 smartphone.

Only one acquisition attempt per eye and per device was permitted. Image acquisition success was defined as successful capture of the Optic Nerve (ON) head within the image frame.

Acquisition time was measured for each device, starting from the moment the imaging system was positioned against the eye until capture of an image judged satisfactory.

For each examination session, the first retinal image was systematically acquired using the reference device (Aurora). The order of use of the four SBFI systems was subsequently determined by computer‐generated randomization prior to each examination (Random.org) in order to minimize sequence bias.

### 2.4 Clinician Validation

A representative expert panel of veterinary ophthalmologists (*n* = 32) was formed to validate and grade all captured fundus images. Panel members included diplomates of the European College of Veterinary Ophthalmologists (ECVO), the American College of Veterinary Ophthalmologists (ACVO), and Members of the French Eye Pannelist (AFEP‐MHOC). The mean professional experience in veterinary ophthalmology was 11 years.

Image evaluation was conducted using an online survey platform (Google Forms, Google LLC, Mountain View, CA, USA). The survey link was distributed via Listserv mailing lists (ECVO and ACVO) as well as through direct communication within the French Eye Pannelists members. Participation was voluntary and responses were recorded anonymously.

For each imaging set, five images obtained from the same eye during the same examination session were presented simultaneously on a single screen. The reference device (Aurora) image was displayed at the top of the panel and explicitly labeled as the reference image. The four smartphone‐based fundus imaging (SBFI) device images were displayed below the reference image in randomized order to minimize positional bias.

To reduce potential bias related to inherent differences in field of view between imaging systems, evaluators were explicitly instructed not to consider field of view or angle of observation when grading image quality. This instruction was provided to ensure that grading focused on image clarity and contrast rather than differences in optical design between devices.

Evaluators were first asked to select the preferred image among the four SBFI systems (forced single‐choice selection). The reference Aurora image was not included in the preference analysis.

Subsequently, evaluators independently graded the image quality of each of the five displayed images (Aurora and the four SBFI systems) using a 10‐point Likert scale, where 1 corresponded to very poor image quality and 10 to excellent image quality.

Each imaging set therefore generated 32 independent evaluations for image quality and SBFI preference.

### 2.5 Statistical Analysis

All statistical analyses were performed using the GraphPad Prism (GraphPad Software, La Jolla, CA, USA). Data distribution was assessed using the D'Agostino–Pearson omnibus normality test. As image quality scores did not follow a normal distribution, non‐parametric tests were used for comparative analyses.

Image quality was analyzed in a two‐step approach. First, each smartphone‐based fundus imaging (SBFI) system was compared with the reference device (Aurora) using pairwise non‐parametric tests. Second, comparisons among the four SBFI systems were performed using the Kruskal–Wallis test followed by Holm‐adjusted post hoc pairwise comparisons.

Acquisition times were compared using the Friedman test for repeated measures across devices, with post hoc multiple comparisons when appropriate.

The effect of mydriasis on image quality was evaluated using paired Wilcoxon signed‐rank tests for each device separately.

Evaluator preference among the four smartphone‐based fundus imaging (SBFI) systems was analyzed using a chi‐squared goodness‐of‐fit test to determine whether vote distribution differed from a uniform distribution.

All tests were two‐tailed, and a probability value of *p* ≤ 0.05 was considered statistically significant.

## 3 Results

### 3.1 Image Acquisition Success

Successful image acquisition was achieved in all animals for all devices under both mydriatic and non‐mydriatic conditions (100% success rate across all imaging systems). All 40 imaging sets were complete and included in the final analysis.

### 3.2 Image Quality Assessment

**FIGURE 2:**
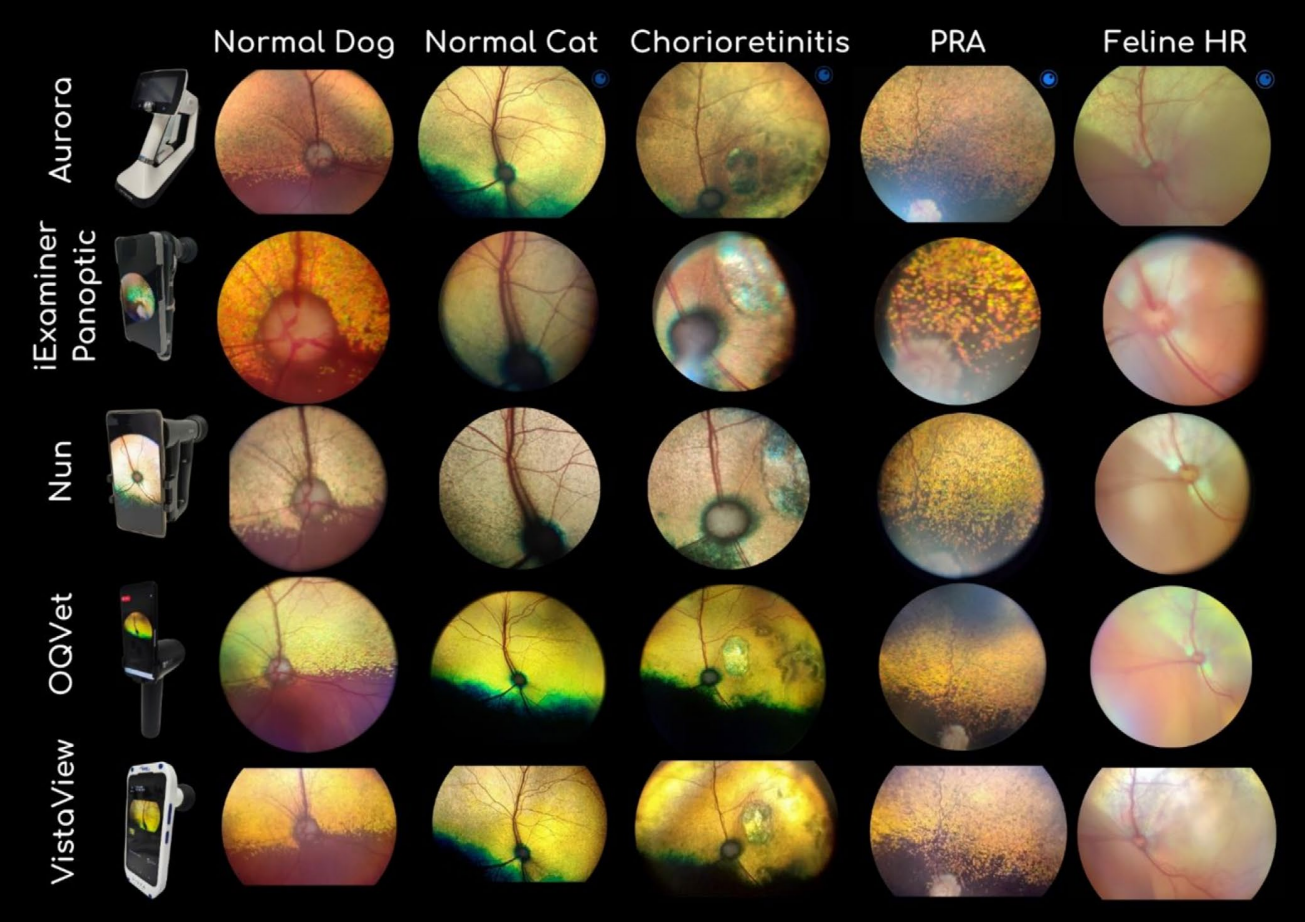
Overview of the reference handheld fundus camera and the four smartphone‐based fundus imaging (SBFI) systems evaluated in this study, with representative retinal images acquired using each device. Devices are displayed by row (Aurora, iExaminer Panoptic, NUN, OQVet, and VistaView), and clinical conditions are presented by column, including normal dog, normal cat, chorioretinitis, progressive retinal atrophy (PRA), and feline hypertensive retinopathy (HR). All images were obtained under standardized imaging conditions. Images were acquired under standardized conditions with illumination adjustments according to species and retinal region.

Image quality scores differed significantly between imaging systems (Kruskal–Wallis test, *p* < 0.0001). Representative fundus images acquired with each device across normal and pathological retinal conditions are shown in Figure 2.

**FIGURE 3:**
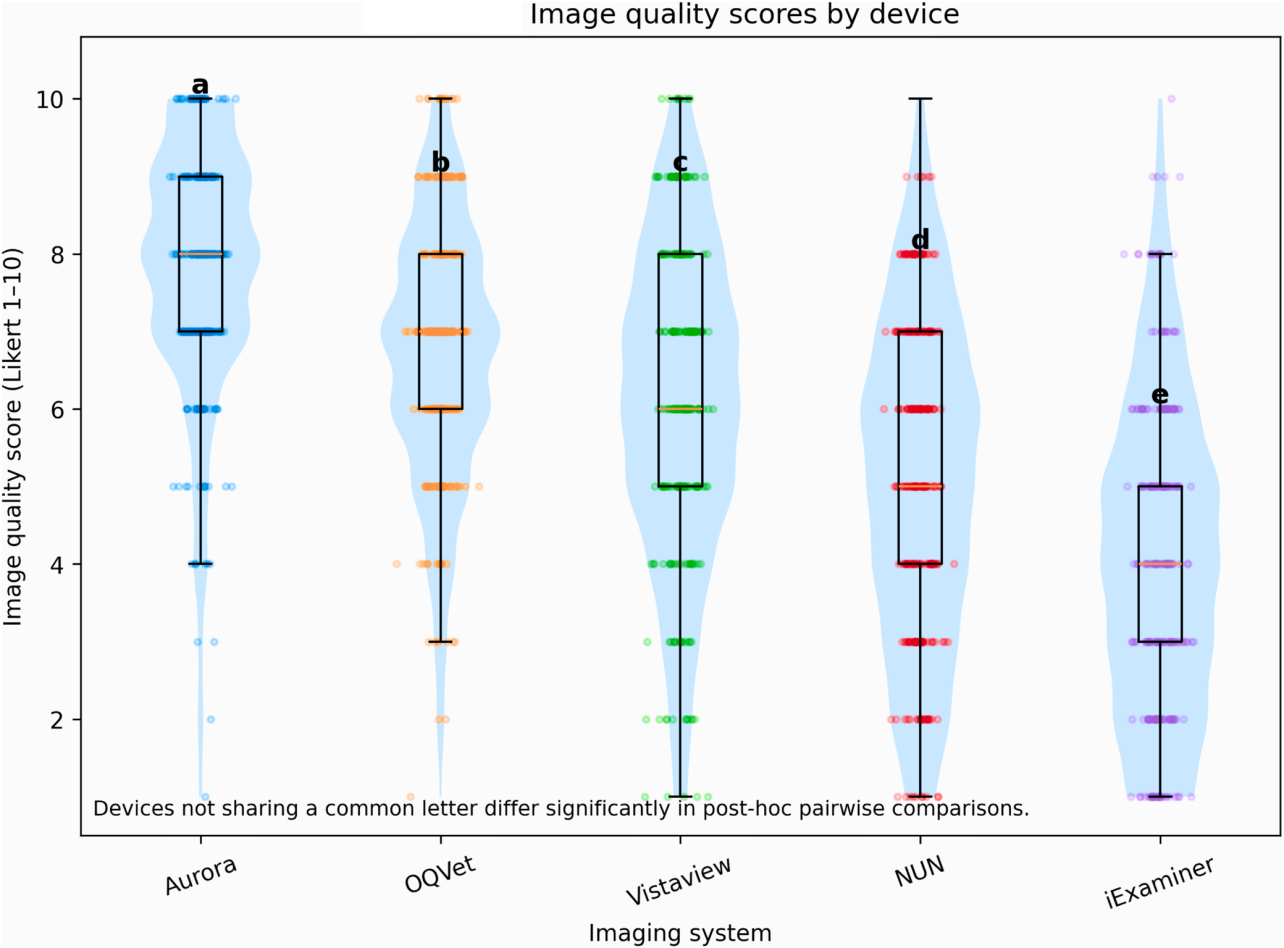
Distribution of image quality scores across imaging systems. Distribution of image quality scores (Likert scale 1–10) across imaging systems. Violin plots represent score distributions, boxplots indicate the median and interquartile range (IQR), and individual points correspond to evaluator ratings. Image quality scores differed significantly between systems (Kruskal–Wallis test, *p* < 0.0001). Devices not sharing a common letter differ significantly based on Holm‐adjusted post hoc pairwise comparisons.

The distribution of image quality scores is presented in Figure 3 which illustrates score distributions across devices and highlights statistically significant differences using group‐based significance annotations. Detailed statistical comparisons are reported in Table 3, including descriptive statistics (A), pairwise comparisons with the reference device (B), and comparisons among smartphone‐based systems (C). Image quality scores were treated as ordinal variables, and graphical representations should be interpreted accordingly.

**TABLE 3:** Image quality scores and pairwise comparisons across imaging systems.

(A) Descriptive statistics of image quality scores (Likert scale 1–10)		
Device	*n*	Median (IQR)
Aurora	1280	8 (7–9)
OQVet	1280	7 (6–8)
VistaView	1280	6 (5–8)
NUN	1280	5 (4–7)
iExaminer	1280	4 (3–5)

(B) Pairwise comparisons between each smartphone‐based system with the reference device (Aurora)		
Comparison	*p* (Holm)	Significance
Aurora vs. OQVet	< 0.001	***
Aurora vs. VistaView	< 0.001	***
Aurora vs. NUN	< 0.001	***
Aurora vs. iExaminer	< 0.001	***

(C) Pairwise comparisons among SBFI systems		
Comparison	*p* (Holm)	Significance
OQVet vs. VistaView	< 0.001	***
OQVet vs. NUN	< 0.001	***
OQVet vs. iExaminer	< 0.001	***
VistaView vs. NUN	< 0.001	***
VistaView vs. iExaminer	< 0.001	***
NUN vs. iExaminer	< 0.001	***

*Note:* Image quality scores (Likert scale 1–10) for the reference device (Aurora) and smartphone‐based fundus imaging systems. (A) Descriptive statistics are presented as median (interquartile range [IQR]). (B) Pairwise comparisons between each smartphone‐based system and the reference device (Aurora). (C) Pairwise comparisons among smartphone‐based systems. Significance levels: ***p < 0.001 (Holm‐adjusted).

The reference device Aurora achieved the highest image quality scores, with a median score of 8 (interquartile range [IQR] 7–9). Among the SBFI systems, OQVet obtained the highest image quality ratings (median 7, IQR 6–8), followed by VistaView (median 6, IQR 5–8), NUN (median 5, IQR 4–7), and iExaminer (median 4, IQR 3–5).

In pairwise comparisons against the reference device, Aurora scored significantly higher than each SBFI system (all Holm‐adjusted *p* < 0.001).

In the secondary analysis restricted to smartphone‐based systems, image quality also differed significantly between devices. OQVet scored significantly higher than VistaView, NUN, and iExaminer; VistaView scored significantly higher than NUN and iExaminer; and NUN scored significantly higher than iExaminer (Table 3; Figure 3).

When analyzing image quality at the imaging set level, Aurora achieved the highest overall image quality score in 31 out of 40 imaging sets (77.5%). Aurora was outperformed by a smartphone‐based fundus imaging system in 9 out of 40 imaging sets (22.5%). In these cases, Aurora was most frequently outperformed by OQVet (6/40, 15%), and less frequently by VistaView (3/40, 7.5%). Aurora was never outperformed by NUN or iExaminer at the imaging set level.

These findings indicate that while Aurora remains the highest‐performing device in the majority of cases, certain SBFI systems—particularly OQVet—can achieve superior image quality in a subset of examinations.

### 3.3 Evaluator Preference Analysis

Evaluator preferences among the four SBFI systems were markedly non‐uniform. The distribution of preference votes differed significantly from a uniform distribution (chi‐squared goodness‐of‐fit test, *p* < 0.001).

**FIGURE 4:**
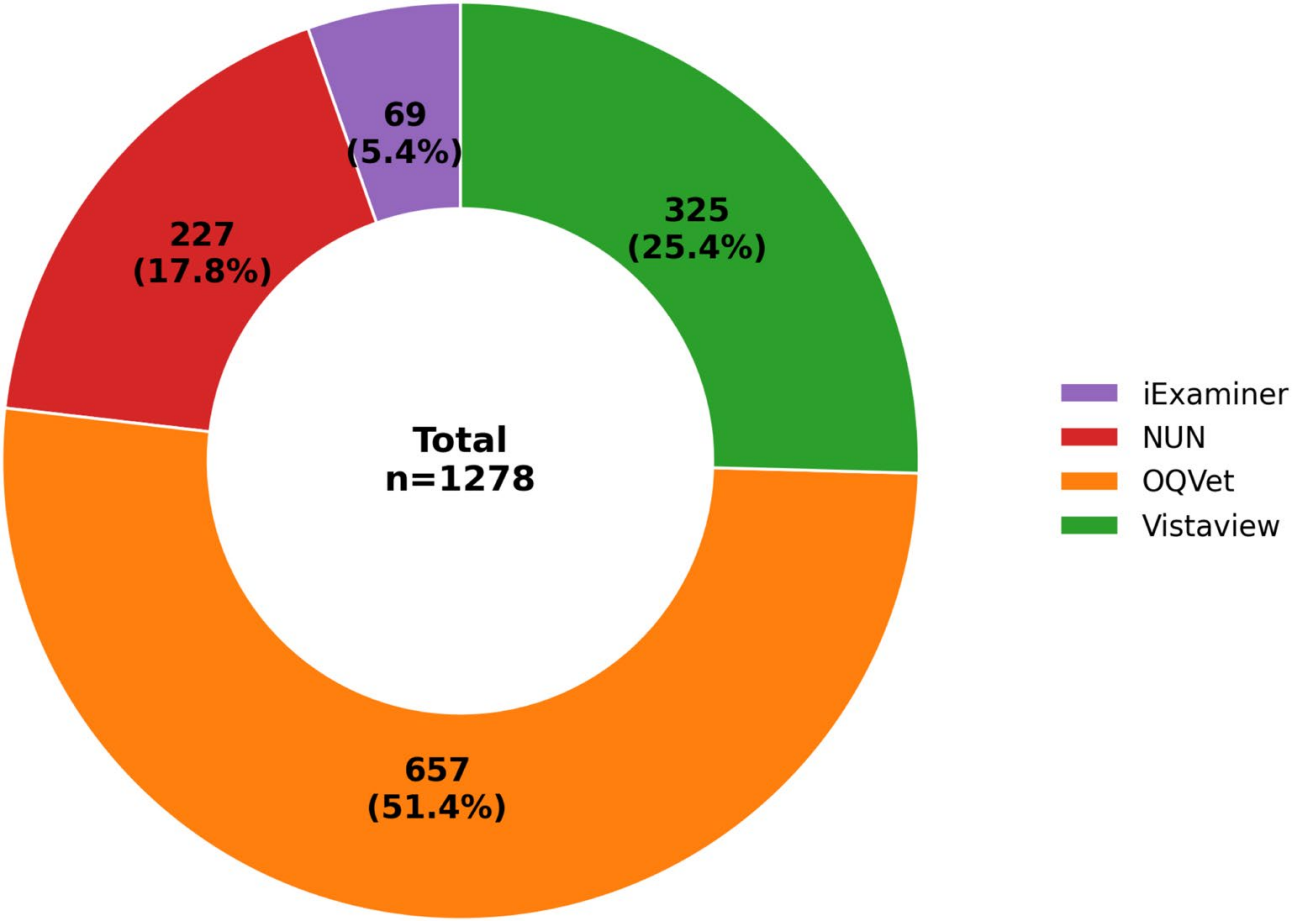
Evaluator preference distribution among smartphone‐based fundus imaging systems. Distribution of evaluator preference votes among smartphone‐based fundus imaging systems. Each segment displays the number of votes (*n*) and corresponding percentage. The overall distribution differed significantly from a uniform distribution (chi‐square goodness‐of‐fit test, *p* < 0.001). Standardized Pearson residuals confirmed significant over‐representation of OQVet preference votes and under‐representation of iExaminer and NUN votes.

OQVet received the highest proportion of preference votes (approximately 50%), followed by VistaView (approximately 25%), NUN (approximately 18%), and iExaminer (approximately 6%). Standardized Pearson residuals confirmed significant over‐representation of OQVet preference votes and under‐representation of iExaminer and NUN votes, whereas VistaView did not differ markedly from the expected distribution (Figure 4), which illustrates the distribution of preference votes across devices.

Evaluator preference among SBFI systems differed between normal and pathological retinas (χ^2^ test, *p* < 0.001), with a marked increase in preference for OQVet in pathological cases (≈60% of votes) compared with normal retinas (≈45%).

### 3.4 Acquisition Time

**FIGURE 5:**
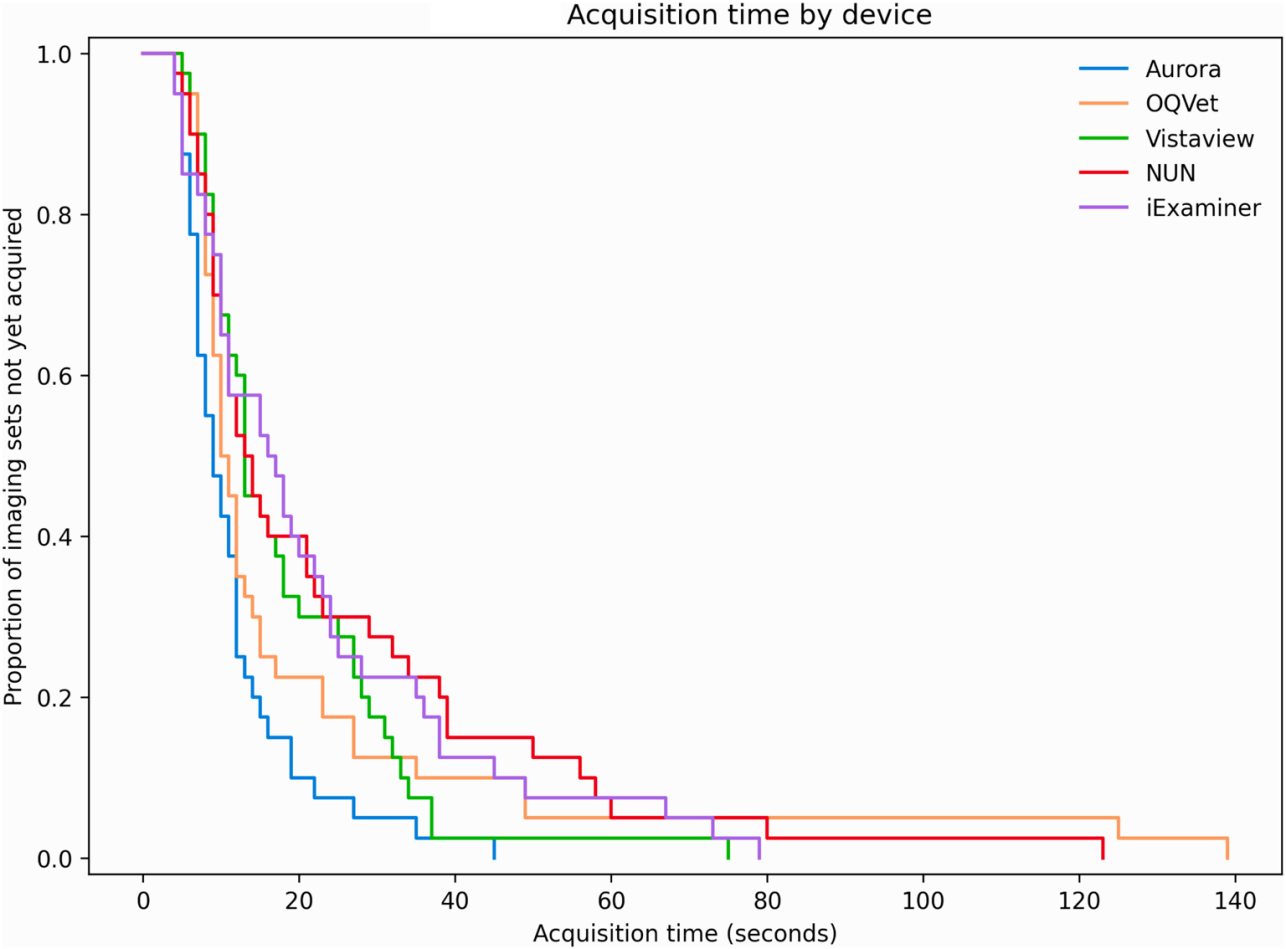
Acquisition time across imaging systems Cumulative distribution of acquisition time (seconds) for each imaging system. Curves represent the proportion of imaging sets not yet acquired over time, allowing comparison of acquisition efficiency between devices. Acquisition times differed significantly between systems (Friedman test, *p* = 0.002).

Acquisition time differed significantly between imaging systems (Friedman test, *p* = 0.002). The distribution of acquisition times is illustrated in Figure 5 using a cumulative representation, allowing comparison of acquisition efficiency across devices. The reference device demonstrated the shortest acquisition time (median approximately 9 s). Among the SBFI systems, OQVet showed shorter acquisition times (median 10–11 s) compared with VistaView and NUN (median 13–14 s), while iExaminer required the longest acquisition time (median 16–17 s) (Table 4; Figure 5).

**TABLE 4:** Acquisition time (seconds) across imaging systems.

Device	*n*	Median (IQR)
Aurora	40	9 (7–12.25)
VistaView	40	13 (9.75–27)
OQVet	40	10.5 (8–15.5)
iExaminer	40	16.5 (9.75–27)
NUN	40	13.5 (9–32.5)

*Note:* Data are presented as median (interquartile range [IQR]). Differences between devices were assessed using the Friedman test for repeated measures, reflecting variability in acquisition efficiency across systems.

Pupil dilation did not significantly influence acquisition time.

### 3.5 Effect of Mydriasis on Image Quality

**FIGURE 6:**
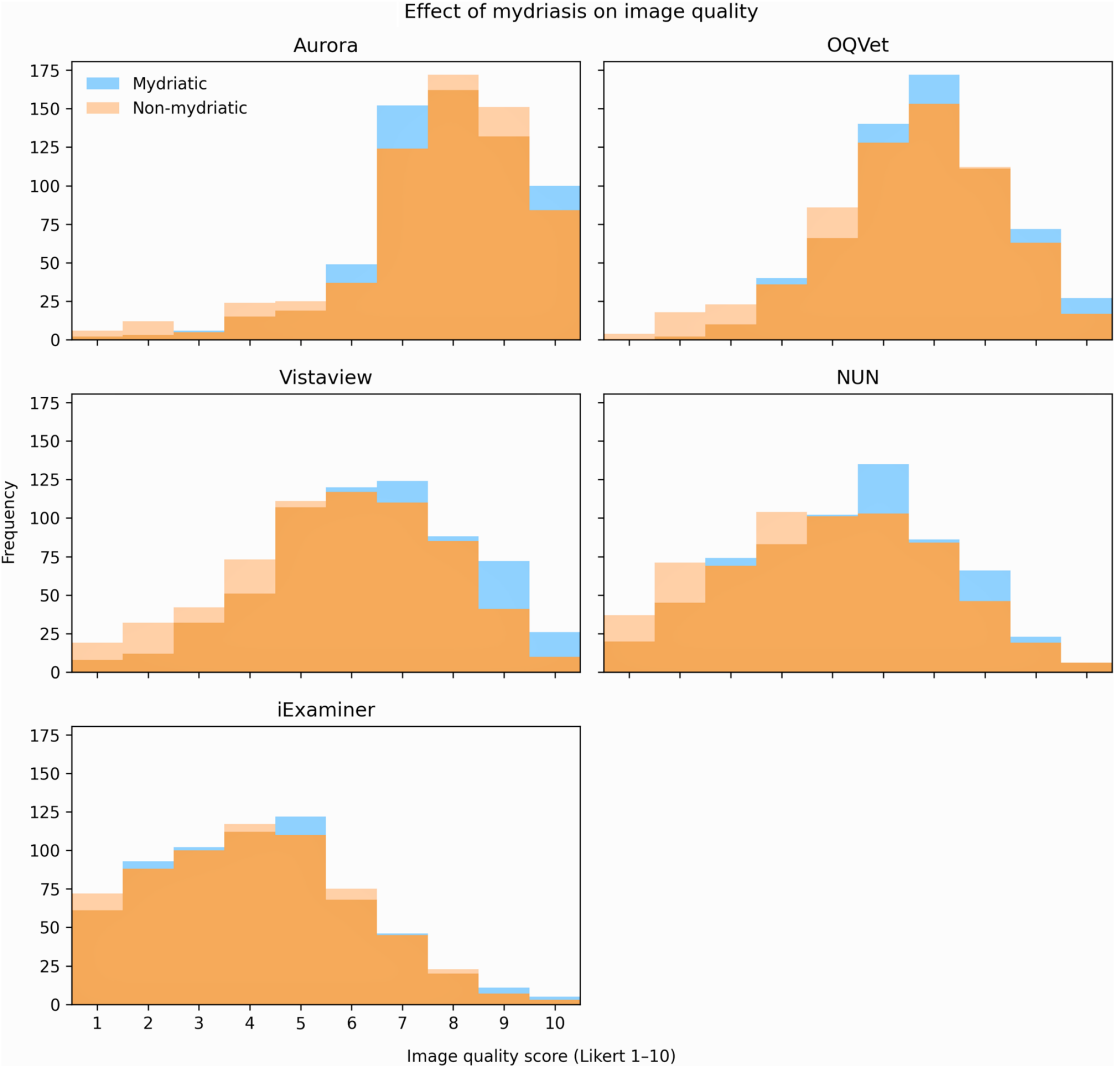
Effect of pupil dilation on image quality scores. Distribution of image quality scores (Likert scale 1–10) under mydriatic and non‐mydriatic conditions for each imaging system. Histograms are superimposed with transparency to facilitate comparison between conditions. No significant differences were observed between mydriatic and non‐mydriatic states across devices (Wilcoxon signed‐rank test).

Paired comparisons between mydriatic and non‐mydriatic conditions did not reveal a statistically significant effect of mydriasis on mean image quality scores for any device (paired Wilcoxon signed‐rank tests, all *p* > 0.05). A non‐significant trend toward higher image quality under mydriatic conditions was observed for VistaView. Results of mydriasis‐related analyses are reported in Table 5 and illustrated in Figure 6, which displays the distribution of image quality scores under mydriatic and non‐mydriatic conditions for each device. Visual inspection of score distributions (Figure 6) supports the absence of a significant effect of mydriasis across devices.

**TABLE 5:** Effect of mydriasis on image quality.

Device	Mydriatic median (IQR)	Non‐mydriatic median (IQR)	*p* (Wilcoxon paired)
Aurora	8 (7–9)	8 (7–9)	> 0.05
OQVet	7 (6–8)	7 (6–8)	> 0.05
VistaView	6 (5–8)	6 (5–8)	> 0.05
NUN	5 (4–7)	5 (4–7)	> 0.05
iExaminer	4 (3–5)	4 (3–5)	> 0.05

*Note:* Paired comparison of mean image quality scores between mydriatic and non‐mydriatic conditions using Wilcoxon signed‐rank tests.

## 4 Discussion

To our knowledge, this is the first study comparing four commercially available smartphone‐based fundus imaging (SBFI) systems under standardized clinical conditions in veterinary ophthalmology. Successful image acquisition was achieved in 100% of animals for all devices under both mydriatic and non‐mydriatic conditions, confirming the technical feasibility of smartphone‐based retinal imaging in dogs and cats. These findings are consistent with previous veterinary and human studies demonstrating that smartphone‐based fundus imaging systems can reliably capture clinically usable retinal images across a wide range of conditions [7, 17, 18].

Despite universal acquisition success, substantial differences in image quality were observed between devices. Among the SBFI systems evaluated, OQVet demonstrated the highest performance, receiving approximately 50% of evaluator preference votes, followed by VistaView (25%), NUN (18%), and iExaminer (6%). These findings highlight significant heterogeneity in performance among commercially available smartphone‐based retinal imaging systems. Similar variability has been reported in human ophthalmology, where differences in optical design, illumination control, and image acquisition architecture significantly influence image quality and diagnostic performance [10, 14, 19].

The optical and illumination characteristics of smartphone‐based systems play a critical role in retinal image quality. These devices rely on reflected illumination, precise optical alignment, and differ substantially in their illumination design (e.g., point, ring, or flood light sources), which directly influences light distribution, contrast, and the occurrence of glare artifacts. In veterinary patients, these effects are further amplified by the presence of the tapetum lucidum, creating a highly reflective environment adjacent to darker non‐tapetal regions. This high‐contrast configuration may result in localized overexposure, reduced contrast, or uneven illumination, as observed in some images, particularly in feline fundi, thereby affecting image interpretability. Similar technical limitations have been previously reported in both veterinary and human smartphone‐based retinal imaging systems [7, 17].

Although imaging was performed under standardized environmental conditions, illumination settings were adjusted by the examiner according to fundus characteristics and were not strictly standardized across all acquisitions. While this reflects routine clinical practice, it may have introduced variability in image appearance and influenced inter‐device comparisons.

However, species‐related differences in fundus reflectivity, particularly the wider tapetal area in cats, may have influenced image appearance and contributed to variability in image quality. This should be considered when interpreting inter‐device comparisons.

Importantly, pupil dilation did not significantly influence image acquisition success or image quality in this study. This finding has important clinical implications, as non‐mydriatic imaging simplifies examination procedures and improves patient comfort while maintaining diagnostic usability. Previous studies have likewise demonstrated that modern smartphone‐based retinal imaging systems can successfully acquire clinically usable retinal images under non‐mydriatic conditions [10, 17].

Smartphone‐based fundus imaging offers several important advantages over conventional retinal imaging systems. These devices are portable, relatively inexpensive, and widely accessible. Cost remains a major barrier to the adoption of retinal imaging equipment in veterinary practice, with recent data identifying equipment cost as one of the primary obstacles to routine ophthalmic examination in companion animal clinics [3]. Smartphone‐based imaging systems provide a practical and affordable alternative, enabling broader access to retinal imaging in general veterinary practice. The OQVet system evaluated in this study currently retails at approximately USD 3500, which remains substantially lower than the cost of most dedicated veterinary retinal imaging systems.

The connectivity and digital integration of smartphones further enhance the clinical utility of these systems by enabling rapid storage, transmission, and remote interpretation of retinal images. Telemedicine applications represent a particularly promising area of development. Studies in human ophthalmology have demonstrated excellent diagnostic reliability using smartphone‐acquired retinal images for teleophthalmology consultations [20, 21]. These capabilities may significantly improve access to veterinary ophthalmology expertise, particularly in geographically isolated regions or general practice settings.

In addition to their clinical applications, smartphone‐based retinal imaging systems offer important advantages for teaching and training. Unlike traditional direct ophthalmoscopy, smartphone imaging allows simultaneous visualization of retinal structures by both instructor and trainee, facilitating education and improving diagnostic learning. Previous studies have demonstrated that smartphone‐based imaging improves teaching effectiveness and enhances understanding of fundoscopic examination [13, 18].

Emerging developments in artificial intelligence (AI) may further expand the clinical utility of smartphone‐based retinal imaging. AI‐assisted diagnostic systems have demonstrated high diagnostic accuracy in detecting retinal diseases using smartphone‐acquired fundus photographs, with reported diagnostic sensitivities and specificities exceeding 85% and 90%, respectively [22]. As smartphone‐based systems continue to improve image quality and accessibility, these technologies may eventually support automated detection of veterinary retinal diseases, including hypertensive retinopathy in cats and inherited retinal degeneration in dogs.

Several limitations of this study should be acknowledged. First, different smartphone models were used depending on device compatibility. Because smartphone‐based imaging systems rely on integrated smartphone cameras, variations in sensor characteristics and image processing may influence image quality. However, this reflects real‐world clinical conditions in which devices are used with their intended smartphone platforms.

Second, all image acquisitions were performed by a single experienced veterinary ophthalmologist. This ensured standardized imaging conditions and eliminated inter‐operator variability, although acquisition performance may differ among less experienced users.

Third, subjective image quality grading, although performed by a large panel of experienced veterinary ophthalmologists, inherently involves some degree of inter‐observer variability. However, the use of 32 independent masked evaluators strengthens the reliability and robustness of the grading process.

Image quality scores were based on ordinal Likert scales; therefore, graphical representations should be interpreted with caution, as these data do not follow a continuous distribution.

Additionally, illumination adjustments made during image acquisition, while reflecting real‐world clinical conditions, may have contributed to variability in image quality and influenced inter‐device comparisons.

Finally, images obtained with smartphone‐based systems were not directly compared with those acquired using a stationary fundus camera. While such systems represent a reference standard in human ophthalmology, their use in routine veterinary practice is often limited by practical constraints, including the need for patient restraint or sedation. Therefore, the present study was designed to reflect real‐world clinical conditions encountered in veterinary ophthalmology.

Overall, this study demonstrates that smartphone‐based fundus imaging systems provide reliable and clinically usable retinal images in veterinary patients. While performance varied between devices, certain systems demonstrated consistently superior image quality and evaluator preference. Smartphone‐based retinal imaging represents a practical, accessible, and cost‐effective solution with significant potential to improve retinal imaging accessibility, facilitate telemedicine applications, enhance clinical education, and support future AI‐assisted diagnostic approaches in veterinary ophthalmology.

## 5 Conclusion

This study demonstrates that smartphone‐based fundus imaging systems provide reliable and clinically usable retinal images in veterinary patients. While performance varies between devices, certain systems—particularly OQVet—show strong potential as practical alternatives for retinal imaging in veterinary practice. There are a large number of different SBFI adapters available to date. All of the presented SBFI approaches are relatively low‐cost compared with conventional retinal imaging devices.

However, with significant discrepancies in diagnostic accuracy for intended disease screening and substantially varying SBFI image quality, more studies on the comparison of SBFI devices and a reference standard for grading of SBFI image quality are warranted. With the swift advancements in smartphone technology (including continuously improving smartphone camera quality) and a variety of devices available, SBFI is as promising as ever to make eye care more accessible and affordable.

The results from this study lay the groundwork for future studies exploring the use of handheld fundus cameras in various disorders and settings. Further studies with larger sample sizes and inclusion of different disorders are required for validation.

## Author Contributions

**Bertrand Michaud:** conceptualization, investigation, funding acquisition, writing – original draft, methodology, validation, visualization, writing – review and editing, software, formal analysis, project administration, data curation, supervision, resources.

## Ethics Statement

All procedures involving animals were approved by the Animal Ethics Committee of VetAgro Sup, Lyon, France (approval number: 2626). The research complied with Directive 2010/63/EU and ARVO guidelines.

## Consent

Written informed consent was obtained from all dog owners prior to inclusion.

## Conflicts of Interest

The author declares no conflicts of interest.

## Data Availability

The data that support the findings of this study are openly available in Figshare at https://doi.org/10.6084/m9.figshare.31467553.
